# Assessing the burden and inequality in the unmet need for hypertension and type 2 diabetes care using a care cascade framework in Tanzania, Lesotho, and South Africa

**DOI:** 10.1017/S1463423626100978

**Published:** 2026-02-25

**Authors:** Denis Okova, Akim Tafadzwa Lukwa, Robinson Oyando, Folahanmi Tomiwa Akinsolu, Abodunrin Olunike, Plaxcedes Chiwire, Charles Hongoro

**Affiliations:** 1 Health Economics Unit, School of Public Health and Family Medicine, Faculty of Health Sciences, University of Cape Town, Cape Town, South Africa; 2 Division of Family Medicine, Family, Community and Emergency Care (FaCE), Faculty of Health Sciences, University of Cape Town, South Africa; 3 Health Economics Research Unit, KEMRI-Wellcome Trust Research Programme, Nairobi, Kenya; 4 Center for Reproduction and Population Health Studies (CeRPHS), Nigerian Institute of Medical Research, Lagos, Nigeria; 5 Department of Public Health, Faculty of Basic Medical and Health Sciences, Lead City University, Ibadan, Oyo State, Nigeria; 6 Department of Biostatistics and Epidemiology, Nanjing Medical University, Jiangning District, Nanjing, Jiangsu, PR China; 7 Department of Health Services Research, CAPHRI Care and Public Health Research Institute, Maastricht University, Maastricht, The Netherlands; 8 Western Cape Department: Health, Western Cape Province, Cape Town, South Africa; 9 School of Health Systems and Public Health (SHSPH), Faculty of Health Sciences, University of Pretoria, Pretoria, Gauteng Province, South Africa; 10 Developmental, Capable and Ethical State, Human Sciences Research Council, Pretoria, Gauteng Province, South Africa

**Keywords:** Care cascade, diabetes, gender disparities, health inequalities, hypertension, non-communicable diseases, socioeconomic status, sub-Saharan Africa, unmet need

## Abstract

**Background:**

The rapidly growing burden of non-communicable diseases (NCDs) in sub-Saharan Africa necessitates a better understanding of access gaps along the care continuum. This study assessed the prevalence and inequality in unmet need for hypertension and diabetes care in Tanzania, South Africa, and Lesotho using a care cascade framework.

**Methods:**

We conducted a cross-sectional analysis of nationally representative Demographic Health Survey (DHS) datasets from Tanzania (2022), South Africa (2016), and Lesotho (2023/24), focusing on adults aged 15 years and older. The study estimated the proportion of adults with hypertension or diabetes who had not been screened, diagnosed, treated, or achieved disease control. Inequality was assessed using Erreygers Normalized Concentration Indices (ENCI), stratified by sex and residence.

**Results:**

Hypertension prevalence was 12.6% (95% CI: 11.7–13.4) in Tanzania, 46.7% (95% CI: 45.0–48.4) in South Africa, and 15.4% (95% CI: 13.8–17.2) in Lesotho. In Lesotho, 9.1% (95% CI: 7.8–10.6) of adults had diabetes. Unmet need was substantial across all countries: 96.5% for hypertension in Tanzania, 84.2% in South Africa, 65.8% in Lesotho, and 84.2% for diabetes in Lesotho. The care cascade framework revealed critical bottle-necks at screening and treatment stages. Inequality analyses revealed strong pro-poor gradients, particularly in screening (ENCIs: Tanzania −0.19, South Africa −0.17, Lesotho hypertension −0.15, Lesotho diabetes −0.24; all *p* < 0.01), with poor men experiencing the most disparities.

**Conclusion:**

Substantial and inequitable gaps exist in hypertension and diabetes care. Policy strategies should prioritize community-based screening, primary care integration, and equity-focused interventions targeting poor men to improve NCD outcomes in the region.

## Introduction

Non-communicable diseases (NCDs), particularly hypertension and diabetes (predominantly type 2, accounting for over 90% of cases), are increasingly significant contributors to the disease burden in Sub-Saharan Africa (SSA) (Mudie *et al.*, 2019; Moyo-Chilufya *et al.*, 2023), driving rising morbidity and premature mortality. For example, an estimated 106 million adults aged 30–79 years in Africa suffer from hypertension, a condition linked to roughly 700,000 deaths annually (World Health Organization, 2022; Ajele *et al.*, 2024). In addition, an estimated 25 million (5%) adults aged 20–79 years have diabetes in Africa, and this number is projected to more than double by 2050 (Ogurtsova *et al.*, 2017).

The growing NCDs burden is fuelled by demographic transitions (Cacciatore *et al.*, 2025), including aging populations (Jane Osareme *et al.*, 2024), rapid urbanization(Juma *et al.*, 2020), dietary shifts (Popkin and Ng, 2022), and sedentary lifestyles, alongside persistent socioeconomic inequalities (Zhou *et al.*, 2025). The global response to NCDs is anchored in the Sustainable Development Goals (SDGs), notably SDG 3.4, which aims to reduce premature mortality from NCDs by one-third by 2030, and SDG 3.8, which promotes Universal Health Coverage (UHC) to ensure equitable access to needed health services, including those for NCDs (Bennett *et al.*, 2020). Despite these commitments, most SSA countries remain off-track to meet these targets due to health system constraints such as inadequate financing for NCDs, poor access and low levels of NCD awareness, among others (Kengne and Mayosi, 2014; Oyando *et al.*, 2025; Savedoff *et al.*, 2025).

National policy responses to NCDs vary across the region. In East Africa for instance, Tanzania has adopted a national NCD strategy (2016–2020) and implemented the Package of Essential Non-communicable Disease Interventions (WHO PEN) at the primary care level (Tanzania Ministry of Health, 2016; World Health Organization, 2020). However, significant gaps persist in diagnosis rates, treatment adherence, and long-term follow-up (Bintabara and Shayo, 2021; Miselli *et al.*, 2021; Tani *et al.*, 2024). In Southern Africa, Lesotho through its national NCD policy (Lesotho Ministry of Health, 2014), has integrated NCD services into its Essential Health Service Package while South Africa’s 2022–2027 NCD strategy includes clinical guidelines and disease registers, though access disparities persist at the province level (South African National Department of Health, 2022). In West Africa, there is a significant unmet need for hypertension along the care cascade in Nigeria (Obagha *et al.*, 2022), and to avert this, the Federal Government of Nigeria adopted a National Multi-sectoral Action Plan (2019–2025) for the prevention of NCDs (Federal Ministry of Health, 2019). Despite the inequitable access and huge unmet need for NCD care, these policy documents suggests that concerted efforts are being put in place by governments across the region to reverse these trends.

A critical barrier to effective NCD control is unmet need in hypertension and diabetes care, which encompasses individuals who have not been screened, remain undiagnosed, are diagnosed-but-untreated, or receive treatment but experience poor disease control (Gabert *et al.*, 2017, Singh *et al.*, 2023; Danladi *et al.*, 2025). In part, the gaps in unmet need are as a result of socioeconomic inequalities in access to and utilization of NCD screening and treatment interventions (Zulu, 2019; Keetile *et al.*, 2021, Oyando *et al.*, 2022). Some studies (Petrella *et al.*, 2007; Gabert *et al.*, 2017; Stokes *et al.*, 2017; Berry *et al.*, 2017; Obagha *et al.*, 2022; Kothavale *et al.*, 2022; Wang *et al.*, 2024), have documented hypertension and diabetes care gaps in at national and subnational levels.

However, there is paucity of evidence on cross-country studies, distribution of unmet needs for NCD care across socioeconomic groups and at a granular level. Thus, this study extends the literature on the prevalence and inequality of unmet need for NCDs in three ways. First, to our knowledge, this is the first multi-country study of prevalence and inequalities in unmet needs for NCD care and thus provides insights on nuances that might be missed by single country studies. Second, using the most recent data sets, this study provides evidence on how NCDs unmet need gaps are distributed across socioeconomic groups. Third, we provide disaggregated (by gender and residence) evidence on unmet need that could aid in informing policy responses that minimizes existing inequalities and aligning with the equity objectives of UHC.

To address this evidence gap, this study leverages recent Demographic and Health Surveys (DHS) from Tanzania, Lesotho, and South Africa to assess both the magnitude and distribution of unmet need for hypertension and diabetes care. By applying a standardized care cascade framework encompassing screening, diagnosis, treatment, and disease control, the present study quantifies attrition at each stage of care and identify where the most significant losses occur. Importantly, it examines how these care gaps vary by socioeconomic status using Erreygers normalized concentration indices (ENCI) and concentration curves. By situating findings within the SDG framework, this study contributes to national NCD planning, identifies priority populations for intervention, and advances cross-country learning on integrated chronic care in SSA.

## Methods

### Data

The present study used nationally representative data from the Demographic and Health Surveys (DHS) conducted in Tanzania (2022) Ministry of Health (2022), South Africa (2016) (National Department of Health (NDoH) and Council (SAMRC), 2019), and Lesotho (2023/24) (Ministry of Health [Lesotho] and ICF, 2024). The DHS employs a stratified, two-stage cluster sampling design to collect detailed information on population health and sociodemographic characteristics. These countries were selected based on the availability of biomarker data on blood pressure (all three countries) and glycated haemoglobin (HbA1c) for diabetes assessment (Lesotho only) as well as presence of variables to allow for a cascade of care analysis. It is important to note that while hypertension data were available for all three countries, diabetes biomarker data were only collected in Lesotho during the survey period. Therefore, the diabetes analysis and unmet need assessment for diabetes care is restricted to Lesotho only. This analysis focused on adults aged 15 years and older for whom relevant biomarker and interview data were available.

Beyond data availability, the three settings were purposively selected to provide comparative insight across diverse points of the NCD epidemiological transition in sub-Saharan Africa, consistent with regional priorities and evidence gaps (Mudie *et al.*, 2019; Bennett *et al.*, 2020). South Africa represents an upper-middle-income context with a mature NCD burden and documented hypertension care cascade short-falls despite relatively developed primary care infrastructure (Peer *et al.*, 2013; Berry *et al.*, 2017; Benade *et al.*, 2023), compounded by the intersection of NCDs and HIV (Levitt *et al.*, 2011). Tanzania illustrates a lower-income context undergoing rapid transition, where studies highlight health system readiness constraints and cascade gaps for hypertension and diabetes (Bintabara and Shayo, 2021; Osetinsky *et al.*, 2022; Tani *et al.*, 2024).

Lesotho provides a critical case of a small lower-middle-income country with high HIV prevalence and rising NCD burden (Moyo-Chilufya *et al.*, 2023) importantly, the 2023/24 LDHS included HbA1c measurement, enabling a diabetes cascade not currently available in the other two study countries. Taken together, this comparative design allows us to examine how unmet need manifests across different health system capacities, development stages, and epidemiological profiles, addressing calls for context-specific NCD strategies in Africa (Kengne and Mayosi, 2014; Atun *et al.*, 2017).

For hypertension, respondents were asked whether they had ever had their blood pressure measured and whether they had ever been told by a health professional that they had high blood pressure. Those who reported a prior diagnosis were further asked if they had been prescribed medication and whether they had taken any antihypertensive medication in the past month. Similarly, for diabetes, respondents were asked if they had ever been tested for high blood sugar, whether they had ever been diagnosed with the condition, and, if so, whether they were currently taking medications to manage their blood sugar. These self-reported items were used to define progression through the screening, diagnosis, and treatment stages of the care cascade.

Two analytic samples were constructed, one for hypertension, which included respondents from all three countries (*n* = 13,308 in Tanzania; *n* = 8,679 in South Africa; *n* = 6,434 in Lesotho), and one for diabetes, restricted to Lesotho (*n* = 6,679), where HbA1c data were collected. Participants were included if they consented to blood pressure and/or blood glucose testing and had valid, non-missing measurements. In the blood pressure sample, participants with implausible blood pressure readings (defined as systolic values below 70 mm Hg or above 270 mm Hg, and diastolic values below 50 mm Hg or above 150 mm Hg) were excluded from the analysis, in line with established thresholds for hypertension diagnosis (Berry *et al.*, 2017; National Department of Health (NDoH) and Council (SAMRC), 2019; KNBS and ICF, 2022; Kothavale *et al.*, 2022; Ministry of Health [Lesotho] and ICF, 2024).

To enable construction of the diabetes and hypertension care cascades, additional exclusion criteria were applied. Only individuals who provided information on ever being screened or diagnosed were included. Additionally, among those who reported a prior diagnosis, only participants who provided information on current use of medication to manage blood sugar or blood pressure were included in the analysis. This allowed for the construction of mutually exclusive and exhaustive stages of the care continuum, aligned with methods used in prior cascade analyses (Berry *et al.*, 2017; Stokes *et al.*, 2017).

### Prevalence of hypertension and diabetes

After attaining the final samples, an individual’s last two of three blood pressure measurements were averaged to obtain the final blood pressure reading of each individual. According to prior research (Berry *et al.*, 2017; Kothavale *et al.*, 2022), individuals were classified as hypertensive if their systolic blood pressure (SBP) was 140 mm Hg or higher, their diastolic blood pressure (DBP) was 90 mm Hg or higher, or if they reported using antihypertensive medication within the previous month. Blood pressure was categorized as normal when SBP was below 120 mm Hg and DBP was below 80 mm Hg. Participants with SBP between 120–139 mm Hg or DBP between 80–89 mm Hg, who were not on blood pressure medication, were considered prehypertensive.

It is important to note that while the present study reports prevalence estimates for prehypertension to characterize the overall cardio-metabolic risk profile of the population, only individuals meeting the full hypertension criteria (as defined above) were included in the subsequent care cascade and unmet need analysis. Similarly, for diabetes assessment in accordance with existing research (Stokes *et al.*, 2017), diabetes was defined as having glycated HbA1c levels of 6.5% or higher, or current use of medications to control blood sugar. Individuals with HbA1c values ranging from 5.7% to below 6.5%, who were not on diabetes treatment, were classified as having pre-diabetes. As with hypertension, the pre-diabetes category was used for descriptive prevalence reporting only, while the care cascade analysis was restricted to individuals meeting full diabetes criteria. Among those identified as diabetic, glycaemic control was assessed using an HbA1c threshold of less than 7.0%, consistent with clinical recommendations (American Diabetes Association, 2019).

### Unmet need for hypertension and diabetes care

This study constructed care cascades for hypertension and diabetes to examine unmet need, following established methodologies (Stokes *et al.*, 2017; Berry *et al.*, 2017; Kothavale *et al.*, 2022). Participants with each condition were categorized into five mutually exclusive stages as detailed in Table 1 and Table 2. Table 1 outlines the hypertension care cascade, where individuals were classified based on blood pressure measurements, screening history, diagnosis status, and treatment. Table 2 presents the parallel decomposition for diabetes using HbA1c levels, testing history, diagnosis, and medication use.

**Table 1.** Decomposition of hypertension [25]

Category	SDM (mmHg)	DBP (mmHg)	Blood Pressure check-up history
Unscreened	≥140	≥90	Never tested BP and no reported prior diagnosis
Screened but undiagnosed	≥140	≥90	BP has been tested but no reported prior diagnosis
Diagnosed-but-untreated	≥140	≥90	Reported prior diagnosis but currently not using any medication
Treated but uncontrolled	≥140	≥90	Reported prior diagnosis and currently using medication (controlled)
Controlled	<140	<90	Reported prior diagnosis and currently taking medication (uncontrolled)

**Table 2.** Decomposition of diabetes

Cascade Stage	Definition
Unscreened	HbA1c ≥ 6.5%; never tested for high blood sugar; no prior diabetes diagnosis
Screened but undiagnosed	HbA1c ≥ 6.5%; previously tested for high blood sugar; no prior diabetes diagnosis
Diagnosed-but-untreated	Prior diabetes diagnosis reported; not currently using oral glycaemic medication or insulin
Treated but uncontrolled	Currently using diabetes medication; HbA1c ≥ 7.0%
Treated but uncontrolled	Currently using diabetes medication; HbA1c < 7.0%

#### Definition and measurement of unmet need

To estimate unmet need, the proportion of individuals advancing through each step of the care cascade was analysed using the number of individuals in the subsequent stage as the denominator. Total unmet need was defined as the cumulative share of individuals falling into the first four cascade stages; unscreened, screened but undiagnosed, diagnosed-but-untreated, and treated but uncontrolled. To operationalize this definition for analysis, binary unmet need variables were created where:

- Value = 1: Unmet need (individuals in any of the first four cascade stages)
- Value = 0: No unmet need (individuals who were screened, screened and diagnosed, diagnosed and treated and those treated and achieved disease control)

### Statistical analysis

All analyses were conducted using *STATA version 17* (Stata Corp. Inc. Texas, USA). Weighted descriptive analyses were conducted to characterize the study population in terms of sex, age, residence, education, and socioeconomic status. The prevalence of hypertension and diabetes were then estimated, disaggregated by demographic and SES. Inequality in unmet need for hypertension and diabetes care was assessed using the Erreygers Normalized Concentration Index (ENCI). The ENCI was selected over the standard concentration index because it provides several important advantages for bounded health variables like binary outcomes (unmet need coded as 0 and 1).

The standard concentration index has known limitations when applied to binary health variables, including potential dependence on the mean of the outcome and boundary issues that can affect interpretation (Erreygers, 2009). The ENCI addresses these limitations by ensuring that the index value remains within the [-1, +1] range regardless of the outcome’s mean prevalence, making it particularly suitable for comparing inequality across different populations and health conditions with varying baseline rates (Erreygers, 2009, Wagstaff *et al.*, 2007). This methodology quantifies how a given binary health outcome (unmet need) is distributed across the socioeconomic spectrum (Wagstaff *et al.*, 2007; Erreygers, 2009).The ENCI ranges from –1 to +1, where values closer to –1 indicate a strong concentration of the outcome among the poor (pro-poor inequality), values closer to +1 indicate concentration among the rich (pro-rich inequality), and values around zero suggest an equitable distribution across socioeconomic groups (Wagstaff *et al.*, 2007).

Following the computation of ENCI values, concentration curves were generated to visually represent the distribution of unmet need by wealth quintile. These curves plot the cumulative share of the population ranked by socioeconomic status against the cumulative share of the outcome (Kakwani and Podder, 1973; Wagstaff *et al.*, 2007), allowing for intuitive visual assessment of inequality relative to the line of perfect equality. A curve that lies along the 45-degree line (the line of equality) indicates perfect equality, where the outcome is evenly distributed across all socioeconomic groups. When the curve lies above the line of equality, it suggests that unmet need is disproportionately concentrated among poorer individuals (pro-poor inequality). Conversely, a curve that lies below the line indicates that unmet need is more concentrated among the wealthier population (pro-rich inequality). The degree of deviation from the line of equality reflects the magnitude of the inequality (Kakwani and Podder, 1973; Wagstaff *et al.*, 2007).

### Measurement of socioeconomic status

Socioeconomic status was measured using the DHS wealth index, which employs a consistent methodology across all countries to ensure comparability for cross-national analysis (Rutstein, 2015). The wealth index is constructed using principal components analysis (PCA) based on household ownership of durable assets (e.g., television, refrigerator, bicycle), housing characteristics (e.g., flooring material, roofing type, water source, sanitation facilities), and other wealth-related variables. The DHS Programme applies a standardized approach where:

- The same set of asset variables common across all country surveys is identified
- Principal components analysis is performed on these common variables
- The first principal component is used to generate a continuous wealth score for each household
- Households are ranked by their wealth score and divided into quintiles within each country

This approach produces wealth quintiles that are country-specific but methodologically comparable, where quintile 1 represents the poorest 20% of households and quintile 5 represents the wealthiest 20% of households within each country’s distribution (Rutstein, 2015). The use of within-country relative rankings rather than absolute wealth levels allows for meaningful comparison of socioeconomic gradients in health outcomes across different economic contexts. The present study utilized the pre-constructed wealth quintile variables provided in each DHS dataset, which follow this standardized methodology. This ensured that this examination of socioeconomic inequalities in unmet need was based on consistent, comparable measures of relative household wealth across Tanzania, South Africa, and Lesotho.

## Results

### Descriptive statistics

The final sample for the hypertension analysis included 13,308 individuals in Tanzania, 8,679 in South Africa, and 6,434 in Lesotho. For the diabetes analysis in Lesotho, the final sample comprised 6,679 individuals (Supplementary Table 1). Most participants in all samples were female, except in Lesotho where sex distribution was nearly equal (48.5% male, 51.5% female). Rural residence dominated in Tanzania (65.6%) and Lesotho (58.3%), while urban residence was more common in South Africa (59.7%). The distribution across socioeconomic quintiles was relatively even in all countries.

#### Hypertension prevalence and distribution

Across all three countries, the overall distribution shows a substantial burden of hypertension. In Tanzania, a majority of individuals had normal blood pressure (50.2% [48.8, 51.6]), with prehypertension affecting 37.3% [36.0, 38.3] and hypertension 12.6% [11.7, 13.4]. South Africa had the lowest proportion of normal BP (23.1% [21.6, 24.6]) and the highest proportion of hypertensive individuals (46.7% [45.0, 48.4]), while Lesotho showed intermediate levels (62.9% [61.0, 64.8] normal BP, 21.7% [20.2, 23.2] prehypertension, and 15.4% [13.8, 17.2] hypertension overall) (Supplementary Table 2).

Age gradients in hypertension were evident across all countries. In Tanzania, the prevalence of normal BP was significantly higher among younger individuals, declining from 62.0% [60.2, 63.7] in those aged 15–24 to 28.2% [25.3, 31.4] among 45–49-year-olds, while hypertension was significantly lower in younger groups, rising from 6.4% [5.7, 7.4] to 27.6% [24.3, 31.2]. In South Africa, hypertension prevalence was substantially higher among older age groups, increasing from 21.3% [19.1, 23.8] among 15–24-year-olds to 83.5% [80.6, 86.1] among those aged 65+, as normal BP declined sharply from 42.3% [38.9, 45.7] to 4.6% [3.3, 6.2]. In Lesotho, normal BP was highest among the youngest (79.1% [76.9, 81.2]) and declined to 42.4% [36.0, 49.1] among those aged 50–64, while hypertension was significantly lower in younger groups, rising from 4.9% [3.6, 6.8] to 33.8% [27.6, 40.6] across these age groups (Supplementary Table 2).

Sex differences were also marked. In Tanzania, women had significantly higher prevalence of normal BP (54.9% [53.3, 56.5]) compared to men (42.0% [40.2, 43.9]). Prehypertension was significantly more prevalent among men (46.7% [44.8, 48.5]) than women (31.9% [30.5, 33.4]). Hypertension affected 13.2% [12.1, 14.3] of women and 11.3% [10.3, 12.5] of men. In South Africa, women had significantly higher prevalence of normal BP (26.3% [24.4, 28.3]) than men (18.3% [16.5, 20.2]), though hypertension was similarly high in both groups (47.4% [45.4, 49.4] in women, 45.6% [43.2, 48.1] in men). In Lesotho, women also had significantly higher prevalence of normal BP (65.1% [62.5, 67.7]) compared to men (60.6% [58.0, 63.1]), although both sexes had notable levels of hypertension (16.5% [14.6, 18.7] in women, 14.3% [12.4, 16.4] in men) (Supplementary Table 2).

Examining SES patterns, hypertension was present across all quintiles in each country. In Tanzania, the proportion of individuals with normal blood pressure decreased from 52.2% [49.3, 55.1] in the poorest quintile (Q1) to 47.0% [44.4, 49.5] in the richest quintile (Q5). Correspondingly, hypertension prevalence was significantly higher in wealthier groups, increasing from 10.3% [8.6, 12.3] in Q1 to 17.2% [15.7, 19.3] in Q5, while prehypertension ranged from 35.8% to 39.2% across quintiles. In South Africa, normal blood pressure was reported in 23.9% [21.0, 27.1] of individuals in Q1 and 20.6% [17.8, 23.7] in Q5. Hypertension prevalence was significantly higher among wealthier groups, rising from 45.6% [42.3, 49.0] in Q1 to 52.1% [48.1, 56.0] in Q5. Prehypertension remained relatively consistent across quintiles, ranging from 27.3% to 32.1%. In Lesotho, 67.0% [63.2, 70.6] of individuals in Q1 had normal blood pressure compared to 53.1% [48.7, 57.5] in Q5. Hypertension prevalence was substantially higher among wealthier groups, increasing from 8.1% [6.6, 9.8] in Q1 to 21.2% [18.3, 24.7] in Q5. Prehypertension ranged between 18.4% and 25.7% across the SES distribution (Supplementary Table 2).

#### Diabetes prevalence and distribution in Lesotho

In Lesotho’s total diabetes sample, 9.1% [7.8, 10.6] were diabetic, 16.0% [14.6, 17.6] prediabetic, and 74.9% [72.5, 77.2] had normal HbA1c levels (Supplementary Table 3). Diabetes prevalence increased steadily with age, rising from 6.4% [4.9, 8.3] among individuals aged 15–24 to 18.2% [13.2, 24.6] among those aged 50–64. Correspondingly, the proportion of individuals with normal HbA1c was significantly higher among younger groups, decreasing from 78.9% [75.6, 81.8] in the 15–24 age group to 59.2% [52.2, 65.9] among those aged 50–64. Pre-diabetes also became more prominent in midlife, affecting nearly one in four individuals aged 50–64 (22.6% [17.5, 28.6]).

Sex differences in glycaemic outcomes appeared minimal. Among men, 9.2% [7.7, 10.9] were classified as diabetic and 16.7% [14.7, 18.9] as prediabetic, while women showed similar proportions at 9.0% [7.3, 11.0] and 15.4% [13.7, 17.4], respectively (Supplementary Table 3). In both groups, approximately three-quarters maintained normal HbA1c levels. With respect to SES, the prevalence of diabetes was substantially higher among wealthier groups, increasing from 2.8% [2.0, 3.9] in the poorest quintile to 11.4% [9.0, 14.3] in the richest. Pre-diabetes followed a similar trend, rising from 9.1% [7.4, 11.1] in the lowest SES group to 20.2% [16.7, 24.2] in the highest. Meanwhile, the share of individuals with normal glycaemic levels was significantly higher among poorer groups, declining from 88.1% [85.7, 90.1] in the poorest group to 68.4% [63.2, 73.1] among the wealthiest (Supplementary Table 3).

#### Prevalence of unmet need for hypertension

The total unmet need for hypertension care was extremely high across all three countries. Among individuals with hypertension, unmet need reached 96.5% [95.8, 97.7] in Tanzania, 84.2% [82.5, 85.7] in South Africa, and 65.8% [59.1, 72.0] in Lesotho (Table 3). Sex-specific patterns varied by country. In Tanzania, unmet need for hypertension was similarly high among men (96.3% [94.3, 97.6]) and women (96.6% [94.7, 97.8]). In South Africa, unmet need for hypertension was significantly higher among men (88.6% [86.2, 90.6]) than women (81.3% [79.2, 83.2]). In Lesotho, men with hypertension had significantly higher prevalence of unmet need (72.1% [64.0, 79.0]) than women (61.5% [54.4, 68.1]) (Table 3).

**Table 3.** Unmet need in hypertension care (all three countries) and diabetes care (Lesotho only) stratified by sex, residence type and SES

	Tanzania (hypertension) % [Confidence Interval]	South Africa (hypertension) % [Confidence Interval]	Lesotho (hypertension) % [Confidence Interval]	Lesotho (diabetes) % [Confidence Interval]
**Sex**				
Male	96.26 [94.26,97.58]	88.60 [86.24,90.60]	71.20 [63.97,78.95]	84.23 [72.36,91.60]
Female	96.57 [94.74,97.78]	81.26 [79.15,83.19]	61.45 [54.36,68.09]	84.10 [73.86,90.83]
**Residence type**				
Urban	95.39 [92.76,97.10]	83.09 [80.87,85.09]	73.37 [68.39,77.82]	81.15 [72.82,87.38]
Rural	97.26 [96.19,98.18]	85.82 [83.27,88.04]	58.05 [46.55,68.73]	86.81 [69.60,94.98]
**SES**				
Q1 (Poorest)	97.27 [94.69,98.78]	88.87 [86.11,91.13]	72.90 [63.90,80.34]	95.29 [81.86,98.91]
Q2 (Poorer)	96.63 [93.87,98.44]	86.76 [82.24,90.27]	57.07 [47.93,65.76]	98.22 [94.17,99.47]
Q3 (Middle)	97.28 [94.31,98.80]	82.58 [79.04,85.62]	59.13 [44.82,72.05]	86.23 [70.20,94.33]
Q4 (Richer)	97.80 [95.15,98.89]	83.43 [80.04,86.34]	70.08 [58.09,79.83]	87.66 [72.60,95.01]
Q5 (Richest)	94.75 [93.23,97.49]	79.00 [74.52,82.57]	68.79 [61.43,75.31]	68.68 [53.73,80.54]
**Total**	**96.47 [95.76,97.69]**	**84.16 [82.50,85.68]**	**65.82 [59.05,72.01]**	**84.17 [75.48,90.18]**

*Note: Diabetes biomarker data were only available for Lesotho in the surveys analysed.*

Across all countries, both urban and rural residents exhibited high levels of unmet need for hypertension. In Tanzania, unmet need was 95.4% [92.8, 97.1] in urban areas and 97.3% [96.2, 98.2] in rural areas. In South Africa, the corresponding figures were 83.1% [80.9, 85.1] (urban) and 85.8% [83.3, 88.0] (rural). In Lesotho, urban residents had significantly higher unmet need for hypertension (73.4% [68.4, 77.8]) compared to rural residents (58.1% [46.6, 68.7]) (Table 3). Socioeconomic differences in unmet need for hypertension were evident, particularly in South Africa and Lesotho. In South Africa, unmet need was significantly higher among poorer groups, declining from 88.9% [86.1, 91.1] in the poorest quintile to 79.0% [74.5, 82.6] in the richest. In Lesotho, unmet need for hypertension ranged from 72.9% [63.9, 80.3] in Q1 to 68.8% [61.4, 75.3] in Q5. In Tanzania, unmet need for hypertension remained consistently high across SES quintiles, ranging from 94.8% to 97.8% (Table 3).

#### Prevalence of unmet need for diabetes in Lesotho

Among individuals with diabetes in Lesotho, 84.2% [75.5, 90.2] had unmet need for care (Table 3). Unmet need for diabetes was similar between men (84.2% [72.4, 91.6]) and women (84.1% [73.9, 90.8]). Rural residents had higher unmet need for diabetes (86.8% [69.6, 95.0]) than their urban counterparts (81.2% [72.8, 87.4]). Socioeconomic differences in unmet need for diabetes were substantial, with unmet need declining from 95.3% [81.9, 98.9] in the poorest quintile to 68.7% [53.7, 80.5] in the richest quintile (Table 3).

**Figure 1.**
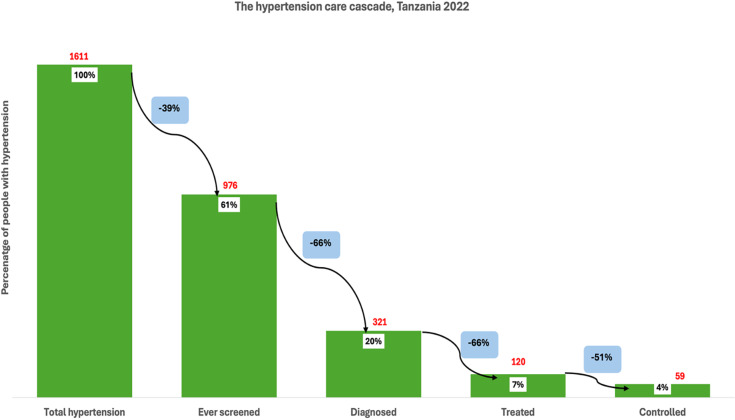
The hypertension care cascade, Tanzania.

**Figure 2.**
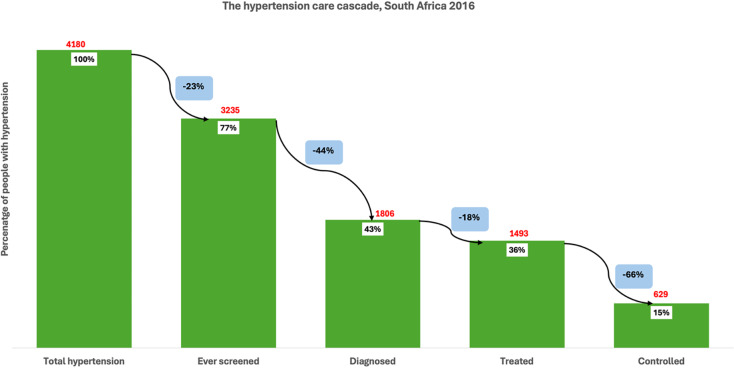
The hypertension care cascade, South Africa.

Among individuals with hypertension in Tanzania (1611), 61% reported that they had ever been screened for high blood pressure, indicating a 39% loss at the first stage of the care cascade **(**Figure 1 **).** Of those with hypertension, only 20% had been diagnosed, representing a further 66% loss from those who had been screened. Among those with hypertension, just 7% were receiving treatment, a 66% loss from the diagnosed group. Finally, only 4% of all individuals with hypertension had their blood pressure controlled, reflecting a 51% loss from those on treatment. Among individuals with hypertension in South Africa (4180), 77% had ever been screened for high blood pressure, indicating a 23% loss at the first stage of the cascade **(**Figure 2 **).** Of the total hypertensive population, 43% had received a diagnosis, reflecting a further 44% loss from those screened. Treatment was reported by 36% of all individuals with hypertension, representing an 18% loss from the diagnosed group. Finally, 15% of individuals with hypertension had their blood pressure controlled, which corresponds to a 66% loss from the treated population.

**Figure 3.**
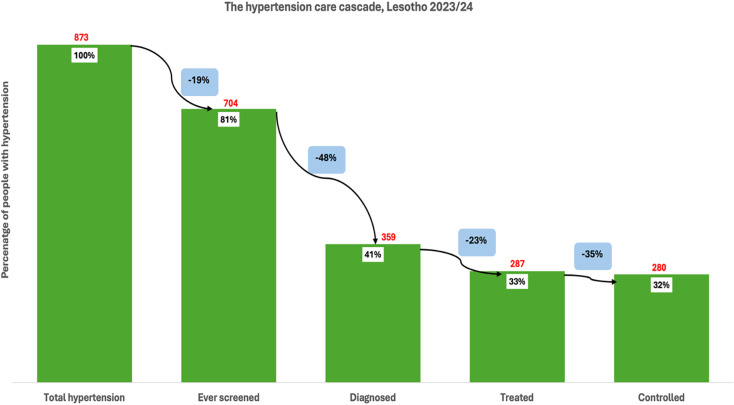
The hypertension care cascade, Lesotho.

**Figure 4.**
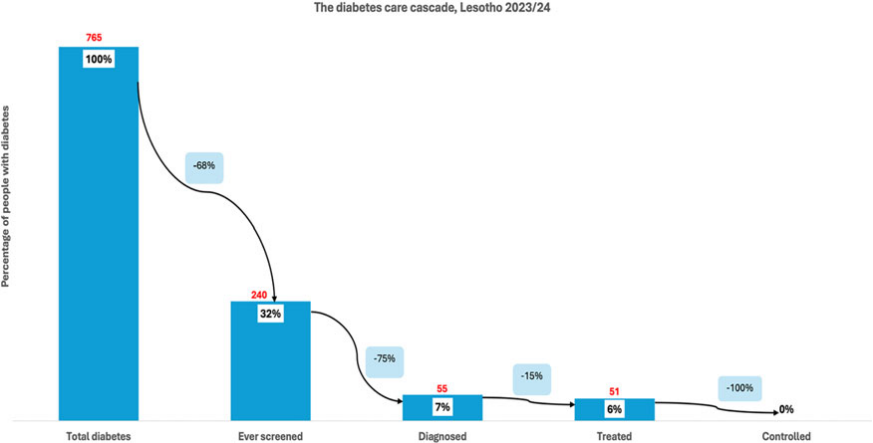
The diabetes care cascade, Lesotho.

**Figure 5.**
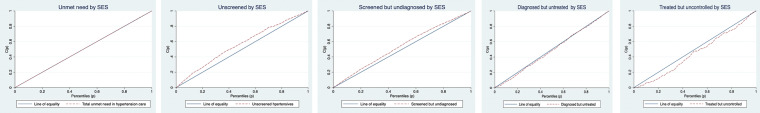
Concentration curves for the hypertension care cascade in Tanzania.

**Figure 6.**
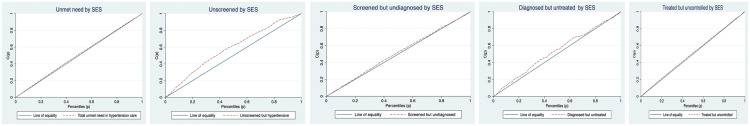
Concentration curves for the hypertension care cascade in South Africa.

**Figure 7.**
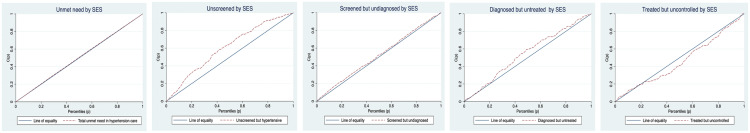
Concentration curves for the hypertension care cascade in Lesotho.

**Figure 8.**
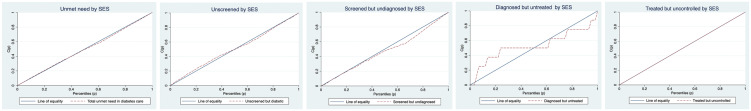
Concentration curves for the diabetes care cascade in Lesotho.

Among individuals with hypertension in Lesotho (873), 81% had ever been screened for high blood pressure, indicating a 19% loss at the first stage of the cascade **(**Figure 3 **).** Of the total hypertensive population, 41% had been diagnosed, reflecting an additional 48% loss from those who were screened. Treatment was received by 33% of individuals with hypertension, marking a 23% loss from those diagnosed. Finally, close to a third (32%) of the total hypertensive population had controlled blood pressure, representing a 35% loss from the treated group. Among individuals with diabetes in Lesotho (765), 32% reported ever being screened for high blood sugar, indicating a 68% loss at the first stage **(**Figure 4 **).** Of the total diabetic population, 7% had received a diagnosis, reflecting a further 75% loss from those screened. Treatment was received by 6% of all individuals with diabetes, representing a 15% loss from the diagnosed group. At the final stage, no one (0%) had achieved glycaemic control, indicating a 100% loss from those on treatment. This highlights significant pro-poor disparities in access to initial screening for hypertension and diabetes across all countries. These results are further corroborated by the concentration curves **(**Figures 5–8 **).**

### Inequality in total unmet need and across the cascade of care

#### Total unmet need

In the overall sample (Table 4), total unmet need for hypertension and diabetes care was disproportionately concentrated among poorer individuals in South Africa and Lesotho (diabetes), though results varied by country and condition. In South Africa, the ENCI for total unmet need was –0.07 (*p* < 0.01), indicating a significant pro-poor gradient of moderate policy relevance. Similarly, Lesotho (diabetes) recorded a strong and statistically significant pro-poor inequality (ENCI = –0.14, *p* < 0.01), representing a substantial gradient that warrants policy attention. In contrast, Tanzania (ENCI = –0.01, *p* = 0.32) and Lesotho (hypertension) (ENCI = –0.01, *p* = 0.89) showed minimal inequality that was neither statistically significant nor substantively meaningful for policy intervention.

**Table 4.** Erreygers normalized concentration indices for the hypertension and diabetes care cascade

Outcome variables	Tanzania			South Africa			Lesotho (Hypertension)			Lesotho (Diabetes)		
	ENCI	p-values	Standard errors	ENCI	p-values	Standard errors	ENCI	p-values	Standard errors	ENCI	p-values	Standard errors
Total unmet need	−0.01	0.32	0.01	−0.07***	0.00	0.01	−0.01	0.89	0.04	−0.14***	0.00	0.03
Unscreened	−0.19***	0.00	0.03	−0.17***	0.00	0.02	−0.15***	0.00	0.03	−0.24***	0.00	0.05
Screened but undiagnosed	−0.19***	0.00	0.03	−0.05	0.11	0.03	−0.06	0.16	0.04	−0.17**	0.04	0.08
Diagnosed-but-untreated	0.06	0.28	0.06	−0.05*	0.06	0.03	−0.07	0.13	0.05	−0.14	0.22	0.11
Treated but uncontrolled	0.14	0.13	0.09	−0.03	0.24	0.03	0.09	0.15	0.05	0.00	0.00	0.00

***p < 0.01, **p < 0.05, * p < 0.1.

#### Unscreened population

The unscreened stage consistently displayed the most significant and substantively important pro-poor inequalities across all countries. ENCI values were –0.19 in Tanzania (*p* < 0.01), –0.17 in South Africa (*p* < 0.01), –0.15 in Lesotho (hypertension) (*p* < 0.01), and –0.24 in Lesotho (diabetes) (*p* < 0.01). These large effect sizes indicate that individuals from lower socioeconomic groups were substantially more likely to remain unscreened, representing a critical equity concern that demands targeted screening interventions for disadvantaged populations (Table 4).

#### Screened but undiagnosed

Among those screened but undiagnosed, pro-poor inequality remained evident with meaningful effect sizes in several settings. Statistically significant gradients with moderate policy relevance were observed in Tanzania (ENCI = –0.19, *p* < 0.01) and Lesotho (diabetes) (ENCI = –0.17, *p* < 0.05). The gradients in South Africa (ENCI = –0.05, *p* = 0.11) and Lesotho (hypertension) (ENCI = –0.06, *p* = 0.16) showed consistent pro-poor patterns with small but potentially meaningful effect sizes that, while not statistically significant, may still indicate systematic barriers to diagnosis among poorer populations that merit monitoring (Table 4).

#### Diagnosed-but-untreated

At the diagnosed-but-untreated stage, patterns were mixed with generally modest effect sizes. In Tanzania, a small, non-significant pro-rich gradient was observed (ENCI = 0.06, p = 0.28), suggesting minimal systematic inequality. In South Africa, the inequality was statistically significant but small in magnitude (ENCI = –0.05, *p* = 0.06), indicating limited policy relevance. In Lesotho, pro-poor inequality of moderate substantive importance was detected for both hypertension (ENCI = –0.07, *p* = 0.13) and diabetes (ENCI = –0.14, *p* = 0.22), with the latter representing a potentially meaningful gradient that may reflect financial barriers to treatment initiation despite lacking statistical significance (Table 4).

#### Treated but uncontrolled

For those treated but uncontrolled, ENCI values were not statistically significant in any setting and generally showed small effect sizes. A positive gradient of moderate magnitude was observed in Tanzania (ENCI = 0.14, *p* = 0.13) and Lesotho (hypertension) (ENCI = 0.09, *p* = 0.15), suggesting potential systematic differences in treatment quality or adherence that, while not definitive, warrant further investigation. South Africa showed a small, non-significant negative gradient (ENCI = –0.03, *p* = 0.24) with minimal policy relevance. In Lesotho (diabetes), the ENCI was 0.00, indicating no meaningful inequality at this stage (Table 4).

#### Sex-stratified inequality patterns

##### Females

Sex-stratified results (Table 5) revealed differences in patterns between women and men. Among women, total unmet need showed significant pro-poor inequality of small to moderate magnitude in South Africa (ENCI = –0.06, *p* < 0.01) and Lesotho (diabetes) (ENCI = –0.11, *p* < 0.05), while the gradient was minimal in Tanzania (ENCI = –0.02, *p* = 0.07) and negligible in Lesotho (hypertension) (ENCI = –0.02, *p* = 0.74). At the unscreened stage, significant pro-poor inequality of moderate importance was observed in Tanzania (ENCI = –0.15, *p* < 0.01) and South Africa (ENCI = –0.13, *p* < 0.01), with smaller but potentially meaningful gradients in Lesotho (hypertension) (ENCI = –0.06, *p* = 0.07) and Lesotho (diabetes) (ENCI = –0.12, *p* = 0.07).

**Table 5.** Sex-stratified Erreygers normalized concentration indices for the hypertension and diabetes care cascade

Outcome variables	Tanzania			South Africa			Lesotho (Hypertension)			Lesotho (Diabetes)		
	ENCI	p-values	Standard errors	ENCI	p-values	Standard errors	ENCI	p-values	Standard errors	ENCI	p-values	Standard errors
**Females**												
Total unmet need	−0.02*	0.07	0.01	−0.06***	0.00	0.02	−0.02	0.74	0.05	−0.11**	0.02	0.04
Unscreened	−0.15***	0.00	0.03	−0.13***	0.00	0.02	−0.06*	0.07	0.03	−0.12*	0.07	0.07
Screened but undiagnosed	−0.20***	0.00	0.04	−0.01	0.75	0.03	−0.07	0.19	0.06	−0.09	0.42	0.11
Diagnosed-but-untreated	−0.01	0.83	0.06	−0.03	0.31	0.03	−0.03	0.59	0.05	−0.35**	0.03	0.15
Treated but uncontrolled	0.03	0.78	0.12	−0.04	0.31	0.04	0.04	0.59	0.07	0.00	0.00	0.00
**Males**												
Total unmet need	0.01	0.58	0.01	−0.08***	0.00	0.02	−0.04	0.44	0.05	−0.18***	0.00	0.05
Unscreened	−0.22***	0.00	0.04	−0.21***	0.00	0.03	−0.27***	0.00	0.05	−0.33***	0.00	0.07
Screened but undiagnosed	−0.15*	0.05	0.08	−0.11***	0.00	0.04	−0.09	0.24	0.07	−0.26**	0.05	0.13
Diagnosed-but-untreated	0.28**	0.02	0.12	−0.11**	0.01	0.04	−0.17*	0.05	0.09	0.03	0.82	0.15
Treated but uncontrolled	0.28*	0.09	0.16	−0.03	0.55	0.05	0.15*	0.07	0.08	0.00	0.00	0.00

***p < 0.01, **p < 0.05, * p < 0.1.

Among screened but undiagnosed women, substantial inequality was found in Tanzania (ENCI = –0.20, *p* < 0.01), while other settings showed no significant gradient. For diagnosed-but-untreated women, a notable pro-poor gradient of large magnitude was observed in Lesotho (diabetes) (ENCI = –0.35, *p* < 0.05), representing a substantial equity concern, with no significant inequality detected in other settings. At the treated but uncontrolled stage, no statistically significant or substantively important inequality was observed for women across any country.

##### Males

Among men, total unmet need was significantly pro-poor with moderate policy relevance in South Africa (ENCI = –0.08, p < 0.01) and substantial importance in Lesotho (diabetes) (ENCI = –0.18, p < 0.01), but minimal in Tanzania (ENCI = 0.01, p = 0.58) and small in Lesotho (hypertension) (ENCI = –0.04, p = 0.44). Pro-poor inequality among unscreened men was consistently strong, statistically significant, and of substantial policy importance across all settings: Tanzania (ENCI = –0.22, *p* < 0.01), South Africa (ENCI = –0.21, *p* < 0.01), Lesotho (hypertension) (ENCI = –0.27, *p* < 0.01), and Lesotho (diabetes) (ENCI = –0.33, *p* < 0.01) (Table 5).

For screened but undiagnosed men, significant pro-poor gradients of moderate to large magnitude were observed in Tanzania (ENCI = –0.15, *p* = 0.05), South Africa (ENCI = –0.11, *p* < 0.01), and Lesotho (diabetes) (ENCI = –0.26, *p* < 0.05). In Tanzania, a statistically significant pro-rich gradient of substantial magnitude emerged at the diagnosed-but-untreated stage (ENCI = 0.28, *p* < 0.05) and persisted into the treated but uncontrolled stage (ENCI = 0.28, *p* = 0.09), suggesting that wealthier men were substantially more likely to receive treatment but remained uncontrolled a pattern that may reflect differential healthcare access or health-seeking behaviours. These later-stage gradients were not statistically significant or substantively important in other countries.

#### Residence-stratified inequality patterns

##### Rural

In rural settings, pro-poor inequality was pronounced at the early stages of the care cascade (Table 6). In Tanzania, unscreened individuals were significantly concentrated among the poor (ENCI = –0.16, *p* < 0.01), and a similar pattern appeared for those screened but undiagnosed (ENCI = –0.15, *p* < 0.01). In South Africa, both unscreened (ENCI = –0.18, *p* < 0.01) and diagnosed-but-untreated individuals (ENCI = –0.07, *p* < 0.05) were disproportionately poor, reflecting inequities in access to and uptake of screening and treatment initiation.

**Table 6.** Residence-stratified Erreygers normalized concentration indices for the hypertension and diabetes care cascade

Outcome variables	Tanzania			South Africa			Lesotho (Hypertension)			Lesotho (Diabetes)		
	ENCI	p-values	Standard errors	ENCI	p-values	Standard errors	ENCI	p-values	Standard errors	ENCI	p-values	Standard errors
**Rural**												
Total unmet need	0.01	0.70	0.01	−0.05**	0.01	0.02	−0.06	0.24	0.05	−0.10**	0.03	0.05
Unscreened	−0.16***	0.00	0.04	−0.18***	0.00	0.03	−0.20***	0.00	0.04	−0.23***	0.00	0.06
Screened but undiagnosed	−0.15***	0.00	0.04	0.00	0.96	0.04	−0.05	0.38	0.06	−0.07	0.62	0.13
Diagnosed-but-untreated	0.06	0.45	0.08	−0.07*	0.05	0.03	−0.15***	0.00	0.06	−0.31*	0.06	0.16
Treated but uncontrolled	0.32**	0.01	0.13	−0.05	0.29	0.04	0.09	0.23	0.08	0.00	0.00	0.00
**Urban**												
Total unmet need	−0.03**	0.01	0.01	−0.07***	0.00	0.02	−0.10**	0.04	0.04	−0.15***	0.00	0.04
Unscreened	−0.14***	0.00	0.04	−0.15***	0.00	0.02	−0.16***	0.04	0.05	−0.25***	0.00	0.07
Screened but undiagnosed	−0.15**	0.05	0.08	−0.08**	0.02	0.03	−0.11*	0.09	0.06	−0.20**	0.05	0.10
Diagnosed-but-untreated	−0.03	0.65	0.06	0.01	0.82	0.04	−0.02	0.77	0.07	0.10	0.17	0.07
Treated but uncontrolled	−0.06	0.57	0.11	−0.04	0.29	0.04	−0.04	0.61	0.08	0.00	0.00	0.00

***p < 0.01, **p < 0.05, * p < 0.1.

In Lesotho, rural populations showed consistently strong pro-poor gradients. Among those with hypertension, the unscreened (ENCI = –0.20, *p* < 0.01) and diagnosed-but-untreated (ENCI = –0.15, *p* < 0.01) groups were concentrated among the poor, while for diabetes, the total unmet need (ENCI = –0.10, p < 0.05), unscreened (ENCI = –0.23, *p* < 0.01), and diagnosed-but-untreated (ENCI = –0.31, *p* < 0.1) populations reflected significant socioeconomic inequality disadvantaging poorer individuals. Tanzania’s ‘treated but uncontrolled’ group showed a rare pro-rich pattern (ENCI = 0.32, *p* < 0.05), suggesting that wealthier individuals were more likely to receive treatment but still fail to achieve control.

##### Urban

Across urban areas, inequalities remained largely pro-poor (Table 6). Total unmet need was significantly concentrated among the poor in all four settings; Tanzania (ENCI = –0.03, *p* < 0.05), South Africa (ENCI = –0.07, *p* < 0.01), Lesotho hypertension (ENCI = –0.10, p < 0.05), and Lesotho diabetes (ENCI = –0.15, *p* < 0.01).

The unscreened population also displayed marked pro-poor inequality: ENCI = –0.14 (*p* < 0.01) in Tanzania, –0.15 (*p* < 0.01) in South Africa, –0.16 (*p* < 0.05) in Lesotho hypertension, and –0.25 (*p* < 0.01) in Lesotho diabetes. Additionally, the ‘screened but undiagnosed’ stage remained pro-poor in Tanzania (ENCI = –0.15, *p* < 0.05), South Africa (ENCI = –0.08, *p* < 0.05), and Lesotho diabetes (ENCI = –0.20, *p* < 0.05).

## Discussion

Using nationally representative DHS data from Tanzania, South Africa, and Lesotho, this study assessed socioeconomic disparities in unmet hypertension and diabetes care across the care cascade. The inequality analysis revealed significant socioeconomic inequalities in access to care, with a pronounced concentration of unmet need among the poor, especially during the screening stage. Gender-disaggregated findings showed men being more disadvantaged compared to women. The discussion is structured around three core insights; systemic health system gaps, socioeconomic disparities and gendered inequities, each linked to country-specific policy frameworks and potential levers for reform.

While the high prevalence of hypertension and diabetes is consistent with the established NCD epidemic (Adeloye and Basquill, 2014; Maimela *et al.*, 2016; Berry *et al.*, 2017), the present study points to a possible systemic failure of the care continuum. The stark disparity in control rates from 4% in Tanzania to 32% in Lesotho points not only to fragmented care but to weakness in patient follow-up and sustained treatment systems, a finding corroborated by earlier studies (Manne-Goehler *et al.*, 2019; Geldsetzer *et al.*, 2019).This cross-country study suggests that the integration of NCD services into pre-existing chronic care platforms is a pivotal strategy for strengthening the care cascade. South Africa’s comparative advantage may be largely attributable to this approach, as evidenced by its integrated chronic disease management (ICDM) model and sequential NCD Strategic Plans (South African National Department of Health, 2013; South African National Department of Health, 2022), which have built upon the infrastructure developed for HIV care (Ameh *et al.*, 2017). This has facilitated more effective screening and linkage to care. Lesotho’s adoption of the integrated WHO PEN package further supports this finding, correlating with its moderate achievements. In contrast, Tanzania’s struggle to achieve comparable outcomes, despite having national guidelines (Laar *et al.*, 2019), highlights the challenges of stand-alone or siloed NCD programmes that lack full integration into primary healthcare. Therefore, the findings advocate for a deliberate policy shift towards integrated chronic disease management as a means of health systems strengthening.

The alarmingly high levels of unmet need identified in this study are not an isolated phenomenon but rather reflect a systemic failure in the management of non-communicable diseases across sub-Saharan Africa. These findings align with a consistent body of evidence from nationally representative and subnational studies, which report similarly critical care gaps for hypertension and diabetes in countries including South Africa, India, and Botswana (Berry *et al.*, 2017; Stokes *et al.*, 2017; Tapela *et al.*, 2020; Kothavale *et al.*, 2022). The recurrence of this pattern across diverse contexts in the region, from Ethiopia to Nigeria and Tanzania (Gelassa *et al.*, 2022; Osetinsky *et al.*, 2022; Zerihun *et al.*, 2024), points to fundamental, shared weaknesses in primary healthcare systems. These include fragmented screening programmes, limited diagnostic capacity, and the inadequate integration of NCD services into routine care (Laar *et al.*, 2019; Gafane-Matemane *et al.*, 2023). Consequently, these results reinforce that current approaches are insufficient and that tackling this crisis requires addressing these foundational health system barriers.

Moreover, this analysis underscores that care deficits are not randomly distributed but are systematically concentrated among socioeconomically disadvantaged groups, thereby exacerbating health inequities. The observed unmet need exhibits pro-poor inequality, particularly pronounced at the screening stage. This pattern is consistent with a substantial body of evidence demonstrating that individuals in lower wealth quintiles are consistently less likely to access essential preventive and diagnostic services (Ricci-Cabello *et al*., 2010; Moody *et al.*, 2016; Oyando *et al.*, 2022; Peng *et al.*, 2025), with the poorest often remaining beyond the reach of such interventions (Seedat, 2015; Sorato *et al.*, 2021).The underlying causes of this disparity are likely multifaceted, rooted in a combination of structural barriers such as geographic inaccessibility, prohibitive out-of-pocket costs, and limited health literacy, which disproportionately affect low-income populations (Kothavale *et al.*, 2022; Osetinsky *et al.*, 2022; Amarteyfio *et al.*, 2024). Collectively, these findings suggest that existing health systems may inadvertently perpetuate the exclusion of the most vulnerable by failing to adequately target the early stages of the care cascade, where initial losses to follow-up are most acute. Consequently, policy solutions must extend beyond the expansion of treatment access to proactively dismantle inequities at the point of detection. Promising strategies include but not limited to deploying community-based outreach models to bridge geographic and social gaps, integrating routine blood pressure and glucose checks into a wider array of primary health care services, and strengthening social protection mechanisms to mitigate the indirect and direct financial costs associated with screening and diagnosis.

The deeper screening disparities experienced by poor men, as revealed in this study, underscore a critical blind spot in current NCD care strategies. While the value of female-centric health services is undeniable, their predominance has arguably led to a systemic neglect of male engagement in preventive care (Gómez-Olivé *et al.*, 2017; Jalo *et al.*, 2025). The larger pro-poor inequality for men suggests that simply expanding existing clinic-based models will not close this gap, as demand-side barriers like gendered health beliefs and economic responsibilities persist (Everett and Zajacova, 2015; Singh *et al.*, 2017). Therefore, the key implication of our findings is the urgent need for tailored, gender-responsive approaches. Future interventions must innovate beyond the clinic wall through workplace and mobile screening initiatives, actively integrating hypertension and diabetes checks into male-friendly platforms such as occupational health services or community campaigns.

### Policy implications

The findings revealed systemic failures in the hypertension and diabetes care continuum across Lesotho, South Africa, and Tanzania, point to several critical policy priorities. A central imperative is strengthening the care cascade through integrated service delivery, moving beyond stand-alone NCD programmes to embed care within existing primary health care structures. In Tanzania, where the sharpest attrition occurred between diagnosis and treatment, policy efforts should focus on strengthening follow-up systems within PHC facilities and expanding the role of Community Health Workers (CHWs) in patient tracking and medication delivery, including integration into established HIV and maternal health platforms as outlined in Tanzania’s NCD Strategic Plan (Tanzania Ministry of Health, 2016).

For South Africa, where screening coverage is high but control remains low, the priority should shift toward improving treatment adherence and clinical monitoring by implementing differentiated care models under the National Strategic Plan for NCDs (2022–2027), such as task-shifting to nurses and multi-month medication dispensing (South African National Department of Health, 2022).

In Lesotho, where community screening rates are high but diagnosis and treatment coverage are uneven, policy must focus on strengthening diagnostic capacity at lower-level facilities and ensuring a consistent supply of essential NCD medicines through the Essential Health Services Package(Lesotho Ministry of Health, 2014).

Furthermore, efforts to expand NCD care must be proactively designed with a strong equity lens to address the pronounced socioeconomic disparities uncovered, particularly the pro-poor inequality in screening. Without explicit pro-poor policy measures, reforms risk entrenching rather than reducing health inequalities. Solutions must, therefore, extend beyond expanding treatment to dismantle inequities at the point of detection through targeted community-based outreach, the integration of routine checks into diverse PHC contacts, and social protection mechanisms to mitigate costs. Aligning national NCD responses with the WHO Global Action Plan and African Regional NCD Framework (World Health Organization, 2022) is essential to close these equity gaps.

Finally, the screening disparities experienced by poor men underscore a need for gender-responsive strategies. This requires innovating beyond female-centric, clinic-based models by developing male-friendly initiatives, such as workplace and mobile screening, to actively engage a population that current approaches are failing. This strategic redirection toward integration, equity, and gender-responsiveness is essential for strengthening health systems and achieving true equity in NCD detection and care.

### Strengths and limitations

To the best of our knowledge this is one of the few studies to assess unmet need in diabetes and hypertension in SSA, thereby offering regional insight that can inform practice and policy in similar contexts. Another key strength lies in the application of the care cascade framework, which allows for a systematic, stage-wise assessment of health system performance by identifying bottle-necks in screening, diagnosis, treatment, and control of non-communicable diseases. This framework has proven useful in highlighting points of attrition across diverse NCD programmes in LMIC settings (Manne-Goehler *et al.*, 2019). Lastly, a major strength is the integration of socioeconomic inequality metrics with gender disaggregated analyses. This dual approach reveals how intersecting social determinants, particularly poverty and gender, shape access to NCD services and contribute to differential outcomes.

Despite these strengths, several limitations warrant consideration. First, reliance on self-reported information for screening, diagnosis, and treatment may introduce recall and social desirability biases. Participants may under-report or mis-remember prior screening and diagnosis events, particularly for conditions that may have been identified many years earlier. This could lead to mis-classification in the care cascade, potentially under-estimating the proportion in the ‘unscreened’ and ‘screened but undiagnosed’ categories if individuals forget prior healthcare encounters. Conversely, social desirability bias may lead to over-reporting of positive health behaviours such as medication adherence. These measurement errors could affect the precision of our unmet need estimates across all cascade stages.

Second, DHS biomarker data are limited to a single point in time, which may misclassify disease status or control. Hypertension and diabetes measurements taken on a single day may not reflect usual clinical status, as both conditions exhibit daily variability influenced by factors such as stress, recent food intake, or medication timing. Third, while the analysis adjusts for key confounders, unobserved factors such as health-seeking behaviour, provider biases, or regional variation in service availability may also influence outcomes. Lastly, the analytical design is cross-sectional, precluding causal inference regarding the drivers of inequality. While the ENCIs capture the direction and magnitude of socioeconomic gradients, they cannot disentangle the causal mechanisms underlying inequality, such as whether disparities arise from health system barriers, behavioural factors, or structural determinants. Hence, associations reported here should be interpreted as descriptive rather than causal.

## Conclusion

In summary, this study underscores three policy imperatives; strengthening integrated NCD care within primary healthcare systems, embedding gender-responsive strategies to reach men, and closing pro-poor gaps in early care cascade stages through outreach and financial protection. Targeted action on these priorities is essential to prevent deepening inequalities in NCD care as countries advance toward universal health coverage in sub-Saharan Africa.

## Supporting information

Okova et al. supplementary material 1 Okova et al. supplementary material

Okova et al. supplementary material 2 Okova et al. supplementary material

Okova et al. supplementary material 3 Okova et al. supplementary material

## Data Availability

Datasets are publicly available at https://dhsprogram.com/data/available-datasets.cfm (accessed on 20 December 2024).

## Supplementary material

The supplementary material for this article can be found at https://doi.org/10.1017/S1463423626100978
