# Supplementary material for: Assessing the burden and inequality in the unmet need for hypertension and type 2 diabetes care using a care cascade framework in Tanzania, Lesotho, and South Africa
Source: Prim Health Care Res Dev. 2026 Feb 25;27:e28. doi: 10.1017/S1463423626100978 (PMC12951333; doi:10.1017/S1463423626100978)
Supplement: Okova et al. supplementary material 1 — Okova et al. supplementary material [file S1463423626100978sup001.docx]

***Supplementary Table 1; Characteristics of the final analytic sample***

|  | **Tanzania (Hypertension)** | | **South Africa (Hypertension)** | | **Lesotho (Hypertension)** | | **Lesotho (Diabetes)** | |
| --- | --- | --- | --- | --- | --- | --- | --- | --- |
|  | **N** | **% [CI^[[1]](#footnote-1)^]** | **N** | **%[CI]** | **N** | **%[CI]** | **N** | **%[CI]** |
| **Sex** | | | | | | | | |
| Male | 5 722 | 36.24 [35.24,37.07] | 3 460 | 40.40 [39.17,41.64] | 3 145 | 48.54 [47.17,49.92] | 3 303 | 48.58 [47.21,49.94] |
| Female | 7 586 | 63.76 [62.93,64.58] | 5 219 | 59.60 [58.36,60.83] | 3 289 | 51.46 [50.08,52.83] | 3 376 | 51.42 [50.06,52.79] |
| **Age categories** |  | | | | | | | |
| 15-24 | 5 355 | 39.62 [38.60,40.65] | 2 067 | 23.61 [22.34,24.92] | 2 181 | 33.48 [31.67,35.34] | 2 171 | 33.59 [31.78,35.44] |
| 25-34 | 3 775 | 29.29 [28.19,30.43] | 2 054 | 24.26 [23.13,25.85] | 1 831 | 28.22 [26.61,29.89] | 1 809 | 28.10 [26.50,29.76] |
| 35-44 | 2 992 | 22.42 [21.50,23.37] | 1 368 | 16.48 [15.42,17.59] | 1 486 | 23.65 [22.04,25.33] | 1 477 | 23.72 [22.11,25.42] |
| 45-49 | 1 186 | 8.66 [8.07,9.29] | 593 | 6.62 [6.00,7.30] | 565 | 9.12 [8.10,10.24] | 560 | 9.05 [8.07,10.14] |
| 50-64 | - | - | 1 544 | 17.33 [16.17,18.56] | 371 | 5.53 [4.85,6.30] | 369 | 5.54 [4.86,6.31] |
| 65 and above | - | - | 1 053 | 11.50 [10.47,12.62] |  |  |  |  |
| **Residence type** | | | | | | | | |
| Urban | 4 522 | 34.42 [30.15,38.95] | 4 601 | 59.68 [57.35,61.97] | 2 216 | 41.73 [38.74,44.79] | 2 189 | 41.62 [38.60,44.71] |
| Rural | 8 786 | 65.58 [61.05,69.85] | 4 078 | 40.32 [38.03,42.65] | 4 218 | 58.27 [55.21,61.26] | 4 197 | 58.38 [55.29,61.40] |
| **Highest education level** | | | | | | | | |
| No education | 1 801 | 13.86 [12.45,15.39] | 803 | 8.46 [7.56,9.44] | 333 | 3.46 [2.92,4.10] | 333 | 3.05 [2.95,4.14] |
| Primary | 6 640 | 53.91 [52.56,55.26] | 1 741 | 18.94 [17.74,20.19] | 2 236 | 31.14 [28.69,33.70] | 2 224 | 31.16 [28.72,33.71] |
| Secondary or higher | 4 867 | 32.23 [30.57,33.94] | 6 050 | 72.61 [70.99,74.17] | 3 785 | 65.40 [62.68,68.02] | 3 750 | 65.34 [62.63,67.96] |
| **SES** | | | | | | | | |
| Q1 (Poorest) | 1 899 | 15.44 [13.44,17.68] | 1 885 | 21.27 [18.67,24.13] | 1 640 | 15.86 [13.72,18.26] | 1 633 | 15.87 [13.77,18.21] |
| Q2 (Poorer) | 2 292 | 17.64 [15.87,19.56] | 1 890 | 20.79 [18.89,22.82] | 1 306 | 17.79 [15.55,20.27] | 1 302 | 17.89 [15.63,20.39] |
| Q3 (Middle) | 2 836 | 20.05 [18.41,21.79] | 2 015 | 21.66 [19.58,23.90] | 1 255 | 20.80 [18.71,23.05] | 1 245 | 20.84 [18.75,23.09] |
| Q4 (Richer) | 3 041 | 22.63 [20.61,24.79] | 1 728 | 19.07 [16.95,21.38] | 1 200 | 23.72 [20.88,26.80] | 1 191 | 23.77 [20.93,26.85] |
| Q5 (Richest) | 3 240 | 24.25 [21.68,27.01] | 1 191 | 17.21 [14.84,19.85] | 1 033 | 21.84 [18.84,25.17] | 1 015 | 21.64 [18.70,24.91] |
| **Sample size (n)** | **13 308** | | **8 679** | | **6 434** | | **6 679** | |

1. Confidence Interval [↑](#footnote-ref-1)
