# Supplementary material for: Assessing the burden and inequality in the unmet need for hypertension and type 2 diabetes care using a care cascade framework in Tanzania, Lesotho, and South Africa
Source: Prim Health Care Res Dev. 2026 Feb 25;27:e28. doi: 10.1017/S1463423626100978 (PMC12951333; doi:10.1017/S1463423626100978)
Supplement: Okova et al. supplementary material 2 — Okova et al. supplementary material [file S1463423626100978sup002.docx]

***Supplementary Table 2; Blood pressure among participants in Tanzania, South Africa and Lesotho***

|  | **Tanzania** | | | **South Africa** | | | **Lesotho** | | |
| --- | --- | --- | --- | --- | --- | --- | --- | --- | --- |
|  | Normal BP  %[CI^[[1]](#footnote-1)^] | Prehypertension  %[CI] | Hypertension  %[CI] | Normal BP  %[CI] | Prehypertension  %[CI] | Hypertension  %[CI] | Normal BP  %[CI] | Prehypertension  %[CI] | Hypertension  %[CI] |
| **Age categories** | | | | | | | | | |
| 15-24 | 61.95[60.20,63.67] | 31.64[30.31,33.61] | 6.41[5.69,7.35] | 42.28[38.90,45.73] | 36.41[33.72,39.18] | 21.32[19.06,23.77] | 79.14[76.89,81.22] | 16.00[14.07,18.14] | 4.87[3.55,6.78] |
| 25-34 | 49.95[47.75,52.16] | 38.35[36.36,40.39] | 11.69[10.36,13.24] | 30.00[27.26,32.90] | 37.94[35.29,40.65] | 32.06[29.07,35.20] | 63.60[60.49,66.66] | 25.07[22.27,28.09] | 11.33[9.54,13.41] |
| 35-44 | 38.45[35.98,40.98] | 43.06[40.88,45.27] | 18.49[16.75,20.37] | 18.98[15.91,22.49] | 33.41[30.35,36.62] | 47.61[44.06,51.18] | 52.93[49.52,56.32] | 24.53[21.97,27.29] | 22.53[19.89,25.42] |
| 45-49 | 28.21[25.25,31.38] | 44.21[40.75,47.72] | 27.58[24.28,31.16] | 12.32[9.39,16.01] | 29.88[25.05,35.22] | 57.79[52.54,62.88] | 39.61[33.51,46.05] | 23.11[19.02,27.79] | 37.28[30.23,44.39] |
| 50-64 | - | - | - | 7.39[5.82,9.34] | 20.43[18.59,23.39] | 72.18[69.44,74.77] | 42.42[35.97,49.14] | 23.80[19.02,29.34] | 33.78[27.55,40.63] |
| 65 and above | - | - | - | 4.56[3.32,6.23] | 11.90[9.69,14.25] | 83.54[80.62,86.10] | - | - | - |
| **Sex** | | | | | | | | | |
| Male | 42.01[40.17,43.86] | 46.66[44.83,48.50] | 11.33[10.27,12.50] | 18.30[16.53,20.20] | 36.06[34.18,37.98] | 45.64[43.24,48.06] | 60.60[58.03,63.12] | 25.13[22.99,27.40] | 14.26[12.36,16.40] |
| Female | 54.93[53.32,56.53] | 31.91[30.45,33.41] | 13.16[12.07,14.34] | 26.30[24.44,28.25] | 26.34[24.87,27.87] | 47.36[45.38,49.39] | 65.11[62.48,67.65] | 18.38[16.42,20.51] | 16.52[14.55,18.68] |
| **Residence type** | | | | | | | | | |
| Rural | 51.07[49.33,52.80] | 37.92[36.37,39.49] | 11.01[10.02,12.09] | 27.65[24.88,30.60] | 25.60[23.75,27.54] | 46.75[43.98,49.54] | 66.94[64.71,69.10] | 20.02[18.19,22.00] | 13.04[10.88,15.54] |
| Urban | 48.67[46.73,50.62] | 35.99[34.22,37.80] | 15.34[13.94,16.86] | 25.34[22.90,27.96] | 26.87[24.74,29.11] | 47.79[45.14,50.45] | 57.31[53.76,60.79] | 23.93[21.63,26.39] | 18.76[16.39,21.38] |
| **Highest education level** | | | | | | | | | |
| No education | 49.78[46.58,52.98] | 38.24[35.11,41.47] | 11.98[10.27,13.93] | 8.30[5.88,11.61] | 16.95[13.07,21.69] | 74.74[69.83,79.10] | 60.60[53.27,67.48] | 26.99[21.20,33.69] | 12.41[8.35,18.04] |
| Primary | 47.98[46.37,49.60] | 38.66[37.18,40.17] | 13.36[12.21,14.59] | 14.97[12.43,17.93] | 24.15[21.02,27.57] | 60.88[56.99,64.64] | 62.68[59.26,65.98] | 22.49[20.19,24.97] | 14.83[12.53,17.47] |
| Secondary or higher | 54.23[52.25,56.30] | 34.48[32.55,36.46] | 11.29[10.12,12.58] | 31.69[29.32,34.15] | 28.29[26.41,30.24] | 40.03[37.78,42.31] | 63.41[60.77,65.98] | 21.08[19.13,23.17] | 15.51[13.54,17.71] |
| **SES** | | | | | | | | | |
| Q1 (Poorest) | 52.19[49.31,55.05] | 37.51[34.86,40.23] | 10.31[8.62,12.27] | 23.91[21.02,27.06] | 30.48[27.88,33.21] | 45.61[42.28,48.99] | 66.96[63.15,70.56] | 24.79[21.78,28.46] | 8.07[6.62,9.80] |
| Q2 (Poorer) | 51.38[48.86,53.89] | 39.21[36.87,41.60] | 9.41[8.22,10.76] | 25.79[23.29,28.45] | 31.89[29.46,33.43] | 42.32[39.16,45.54] | 70.22[66.67,73.53] | 18.44[15.88,21.32] | 11.34[9.24,13.85] |
| Q3 (Middle) | 51.29[48.91,54.14] | 38.25[35.80,40.75] | 10.46[9.12,11.97] | 22.31[19.41,25.50] | 32.05[26.69,34.50] | 45.83[42.33,49.36] | 64.41[60.71,67.94] | 19.49[16.73,22.57] | 16.11[12.75,20.14] |
| Q4 (Richer) | 50.63[47.37,53.38] | 36.21[34.56,39.99] | 13.15[10.75,14.23] | 22.26[19.10,25.78] | 28.88[26.35,31.54] | 49.44[45.99,52.89] | 62.48[58.70,66.11] | 20.05[17.42,22.96] | 17.48[14.75,20.82] |
| Q5 (Richest) | 46.95[44.44,49.48] | 35.83[33.01,37.04] | 17.22[15.72,19.28] | 20.59[17.77,23.72] | 27.34[24.52,30.36] | 52.07[48.09,56.02] | 53.12[48.70,57.50] | 25.67[22.28,29.38] | 21.20[18.28,24.71] |
| **Total**  **Absolute numbers** | **50.24[48.98,51.60]**  **6,548** | **37.26[36.00,38.32]**  **5,149** | **12.55[11.68,13.37]**  **1,611** | **23.05[21.62,24.58]**  **1,921** | **30.27[29.09,31.07]**  **2,578** | **46.67[44.97,48.37]**  **4,180** | **62.92[61.02,64.79]**  **4,044** | **21.65[20.19,23.19]**  **1,517** | **15.42[13.84,17.15]**  **873** |

1. Confidence Interval [↑](#footnote-ref-1)
