# Supplementary material for: Assessing the burden and inequality in the unmet need for hypertension and type 2 diabetes care using a care cascade framework in Tanzania, Lesotho, and South Africa
Source: Prim Health Care Res Dev. 2026 Feb 25;27:e28. doi: 10.1017/S1463423626100978 (PMC12951333; doi:10.1017/S1463423626100978)
Supplement: Okova et al. supplementary material 3 — Okova et al. supplementary material [file S1463423626100978sup003.docx]

***Supplementary Table 3; Diabetes among participants in Lesotho***

|  | **Normal HbA1c**  **% [Confidence Interval]** | **Prediabetes**  **% [Confidence Interval]** | **Diabetes**  **% [Confidence Interval]** |
| --- | --- | --- | --- |
| **Age categories** | | | |
| 15-24 | 78.88 [75.61,81.81] | 14.71 [12.54,17.18] | 6.41 [4.92,8.33] |
| 25-34 | 79.97 [76.83,82.78] | 14.75 [12.67,17.11] | 5.27 [3.96,6.99] |
| 35-44 | 69.20 [65.12,72.99] | 17.83 [15.34,20.61] | 12.98 [10.43,16.03] |
| 45-49 | 68.80 [62.85,74.18] | 16.28 [12.51,20.92] | 14.92 [10.61,20.58] |
| 50-64 | 59.23 [52.23,65.87] | 22.57 [17.49,28.61] | 18.20 [13.18,24.58] |
| 65 and above | - | - | - |
| **Sex** | | | |
| Male | 74.14 [71.25,76.84] | 16.68 [14.67,18.90] | 9.18 [7.74,10.88] |
| Female | 75.59 [72.83,78.16] | 15.43 [13.69,17.35] | 8.97 [7.32,10.96] |
| **SES** | | | |
| Q1 (Poorest) | 88.08 [85.72,90.09] | 9.12 [7.44,11.14] | 2.80 [1.98,3.94] |
| Q2 (Poorer) | 78.38 [74.39,81.89] | 13.60 [11.24,16.36] | 8.02 [5.87,10.87] |
| Q3 (Middle) | 72.82 [68.52,76.74] | 16.91 [13.81,20.53] | 10.27 [8.00,13.10] |
| Q4 (Richer) | 71.20 [66.66,75.34] | 17.92 [15.12,21.10] | 10.88 [8.05,14.56] |
| Q5 (Richest) | 68.37 [63.19,73.14] | 20.2 2[16.74,24.22] | 11.40 [9.03,14.30] |
| **Total**  **Absolute numbers** | **74.89 [72.46,77.17]**  **4,962** | **16.04 [14.59,17.60]**  **952** | **9.07 [7.79,10.55]**  **765** |
